# An Efficient Processing Strategy to Improve the Flavor Profile of Egg Yolk: Ozone-Mediated Oxidation

**DOI:** 10.3390/molecules28010124

**Published:** 2022-12-23

**Authors:** Bao Chen, Yi Sun, Haobo Jin, Qi Wang, Zhe Li, Yongguo Jin, Long Sheng

**Affiliations:** National Research and Development Center for Egg Processing, College of Food Science and Technology, Huazhong Agricultural University, Wuhan 430070, China

**Keywords:** egg yolk, ozone, volatiles, fatty acid

## Abstract

This study investigated the effect of ozone treatment on egg yolk volatiles and fatty acids. The composition and content of volatile substances and the fatty acid content of the egg yolk were changed significantly after ozonation. With proper ozone treatment (30 min), the aldehyde content in the egg yolk increased from 78.08% to 94.63%, and the relative content of dibutyl amine decreased from 1.50% to 0.00%. There were no significant differences among the types of fatty acids in the egg yolks after being treated with ozone, but there were differences in their relative contents. The results of SDS-PAGE showed no significant difference in yolk protein composition and contents among the groups. SEM results showed that moderate ozone treatment (20 min and 30 min) led to a regular and dense network structure of egg yolk. These results provided a theoretical basis for expanding the application of ozone technology in the egg yolk processing industry.

## 1. Introduction

Egg yolks are used in the food processing industry for their nutritional, organoleptic, and functional properties (emulsification and gelation, etc.) because they not only have excellent organoleptic properties but can also be used as an effective emulsifier to stabilize emulsified foods such as mayonnaise, salad dressings, and ice cream [1]. The main nutrients of egg yolk include protein (17.5%), lipids (32.5%), water (48%), minerals (2%), and rich vitamins. Moreover, egg yolk also contains nutrients of high biological value, such as essential fatty acids, phospholipids, and other lipids [2]. The lipids contained in it are mainly classified as triglycerides (65%), phosphatidylcholine (26%), phosphatidylethanolamine (3.8%), lysophosphatidylcholine (0.6%), cholesterol (4%), and sphingomyelin (0.6%) [3].

Volatiles and fatty acid-related studies of egg yolk play an important role in the development of egg yolk products. Volatiles are valued flavor perception parameters, which help to assess the potential consumer acceptance of egg yolk products [4]. In addition, egg yolk, as a complex food system, should be considered for the improvement of its functional properties while keeping fatty acids unaffected to the maximum extent possible. The egg yolk is rich in fatty acids, and a study has shown that the type and proportion of fatty acids play a crucial role in human health [5].

The modification techniques of egg yolk mainly include enzymatic, chemical, and physical modification, and nowadays phospholipase modification is the most popular one. However, these modification methods have certain limitations such as environmental pollution, high cost, and difficult industrialization [6]. Ozone has been applied as a cold sterilization technology in several fields, such as food surface sterilization and modification of cereal proteins and starches, and has great potential in the application of egg yolk modification [7]. Ozone technology is more efficient and cleaner than other technologies, and ozone is easier to produce industrially at a lower cost. 

In its normal state, ozone appears colorless at relatively low concentrations, and when its concentration increases by more than 15% it takes on a light blue color, which is an isotope of oxygen but is about 10 times more soluble than oxygen in water. It has been approved by the U.S. Food and Drug Administration as a powerful oxidant and as a safe disinfectant in direct contact with food [8]. Additionally, as a powerful and highly reactive oxidant, it is more widely used in the field of cereal protein and starch modification [9], where ozone is directly or indirectly involved in oxidation through decomposition and catalysis. Researchers have established a basic scientific explanation of the mechanism of action on proteins during the ozone oxidation reaction [10]. Several studies have also shown that reactive oxygen species (ROS) induced oxidative stress in proteins and were a major cause of chemical degradation of foods [11]. Large amounts of ROS can further trigger oxidative damage to proteins by oxidizing lipids [12]. This approach might cause some oxidative modifications of proteins, as well as oxidative degradation of essential amino acids such as histidine, tryptophan, tyrosine, methionine, and cysteine. The oxidation of ozone can affect cellular constituents, including phospholipids, proteins, and other macromolecular compounds in cell membranes, generating free radicals and oxidizing fatty acids and polyunsaturated fatty acids to form peroxides [13]. Some researchers found a significant increase in the content of beneficial fatty acids (linoleic and oleic acids) as well as an increase in the proportion of ω-6 to ω-3 fatty acids after ozone treatment of chickpea protein [14].

Previously, we studied the effect of ozone-induced oxidation on egg yolk gel properties and emulsification properties, the chicken egg yolk gel was significantly improved, and emulsification performance was greatly improved after ozonation [15,16]. However, ozone oxidation on egg yolk volatiles and fatty acids has been less studied in the past, the changes of volatiles and fatty acids in egg yolk under different ozone treatment times were investigated in this research. The volatile substances in egg yolk were firstly identified by solid-phase microextraction technique and gas chromatography–mass spectrometry, and comparative analysis, principal component analysis, and cluster analysis were performed. After that, the fractions and relative contents of the fatty acids in egg yolk were determined by gas chromatography and subjected to principal component analysis and cluster analysis. Finally, the egg yolk protein composition was analyzed by sodium dodecyl sulfate polyacrylamide gel electrophoresis and the microstructure was observed by scanning electron microscopy. In this study, we provide a theoretical basis for exploring the potential value of ozone as a new technology to be applied to egg yolk modification.

## 2. Materials and Methods

### 2.1. Materials

Methanol and hexane were both GC-grade chromatographically pure, obtained from Sinopharm Chemical Reagent Co., Ltd. (Beijing, China). Fatty acid standard samples (GC-grade chromatography pure) from Sigma Company (Saint Louis, MO, USA). Sodium chloride, chloroform, sodium hydroxide, anhydrous sodium sulfate, and boron trifluoride are all analytically pure reagents obtained from Sinopharm Chemical Reagent Co., Ltd. (Beijing, China).

### 2.2. Preparation of Egg Yolk Samples

The fresh chicken eggs laid within 24 h were collected from Jiufengshan Chicken Farm (Wuhan, Hubei, China). The eggs were broken and separated with an egg separator, then the yolks were collected. Lastly, the collected yolk liquid was magnetically stirred at 4 °C for 30 min.

### 2.3. Ozone Treatment

The pretreated egg yolk solution (100 mL) was placed in a beaker and ozonated using an ozone generator (OZ-002, Qingdao Zhongke Sanyang Depuration Equipment, Qingdao, China). One end of the hose was connected to the ozone generator and the other end was passed into the egg yolk mixture. Then, the samples were treated with magnetic stirring at a low speed for 0 min, 10 min, 20 min, 30 min, and 40 min, respectively. The ozone output of the ozone generator was 2 g/h and the ozone concentration was 10–15 g/m^3^.

### 2.4. Preparation of Volatile Compound Samples

The 3 g of egg yolk liquid was taken under different ozone treatment times, respectively, immediately filled in a 20 mL headspace bottle, then purged with nitrogen for 1 min, capped and placed in a matching sample tray, and refrigerated at 4 °C for measurement. (Note: the holding time from the injection of the sample into the bottle to the final gas analysis should not exceed 10 h to minimize the effect of oxidation). 

### 2.5. Solid Phase Microextraction (SPME)

The DVB/CAR/PDMS-50/30 μm (Supelco, Bellefonte, PA, USA) triple-coated extraction head was selected as the extraction head for extracting the flavor substances from the samples. According to the manufacturer’s requirements, the extraction head was first placed in the gas chromatography–mass spectrometer (7000D, Agilent, CA, USA) inlet and aged at a temperature of 280 °C for 60 min, and then removed and set aside. The extraction vial was removed from the sample tray, 6 mL of saturated saline and a magnetic stirrer were added, the cap was placed in a 45 °C constant temperature water bath (R301, Gongyi, China) with magnetic stirring, and incubated for 15 min before the extraction head was inserted and the extraction fibers were extended to expose the headspace area. After headspace extraction for 40 min, the extract was removed from the vial and placed in the gas chromatography–mass spectrometer inlet for desorption at a temperature of 250 °C for 2 min.

### 2.6. Gas Chromatography–Mass Spectrometer (GC-MS)

The volatile substances were identified by Agilent GC-MS with the following GC and MS conditions.

Gas chromatographic conditions: the column was an HP-5MS (5% phenyl) methylsiloxane capillary (30 m × 0.25 mm, I.D. × 0.25 μm; Restek; Bellefonte, PA, USA) with high-purity helium (99.999% pure) as carrier gas at a flow rate of 1 mL/min; the inlet temperature was 250 °C and the resolution time was 2 min. The programmed ramp-up conditions were an initial temperature of 40 °C, holding for 10 min, ramping up to 200 °C at 5 °C/min; ramping up to 250 °C at 20 °C/min, holding for 5 min; no splitting.

Mass spectrometry conditions: the ionization mode was the EI source, the transmission line and ion source temperatures were set to 280 °C and 230 °C, respectively, and the electron bombardment mass spectrometry voltage was 70 eV. The mass spectrometry scan amplitude was 25 to 550 amu, and the scan rate was 0.2 s/scan.

### 2.7. Pre-Treatment and Oil Extraction of Fatty Acid Determination Samples

The egg yolks were weighed 4 g in beakers at different ozone treatment times, and chloroform–methanol solution with a volume ratio of 2:1 was added to the centrifuge tubes with a 10 mL pipette in a fume hood. The egg yolks and reagents were mixed thoroughly and then sealed with plastic wrap and placed in an ultrasonic cleaner for 10 min. In a fume hood, the mixture was filtered through filter paper and the filter residue removed, and 2 mL of mixture was added to 0.88% NaCl solution with a pipette. The mixture was placed in a refrigerated centrifuge and centrifuged at 3000 rpm for 10 min. The upper clear liquid was aspirated with a disposable rubber-tipped dropper and syringe, including the film at the critical point, leaving the lower yellow liquid. The yellow liquid was poured into a rotary evaporation flask, and the water temperature was set to 45 °C and evaporated for 30–40 min to remove the organic solvent. The raised egg yolk oil was transferred to a 2 mL microcentrifuge tube, filled with nitrogen, sealed with sealing paper, and stored in a −80 °C refrigerator.

### 2.8. Saponification and Methyl Esterification of Egg Yolk Oil

An oil sample of about 20 mg was taken from the prepared in Section 2.7 an placed in a 10 mL centrifuge tube, 2 mL of 0.5 mol/L sodium hydroxide–methanol solution was added, and it was put it into a water bath at 60 °C for 30 min, then cooled to room temperature. A quantity of 2 mL of 14% boron trifluoride methanol solution was added, the centrifuge tube was placed into a 60 °C water bath for 20 min and cooled to room temperature. We added 2 mL of hexane and shook it, then added 2 mL of saturated sodium chloride solution and shook it. The centrifuge tube was placed in a freezing centrifuge and centrifuged at 1000 rpm for 5 min. The upper organic phase in a dry tube was aspirated, and a small amount of anhydrous sodium sulfate was added, shaken, and removed the water. The oil sample mixture was aspirated with a needleless syringe, passed through a 0.22 μm pore-size organic filter membrane, injected into a brown silk-mouthed sealed bottle with 100 µL of internal standard (5.00 mg/mL) added, filled with nitrogen, sealed with sealing paper, refrigerated in a refrigerator, and left to be tested on the machine.

### 2.9. Determination of Fatty Acids

The crude oil samples were detected by GC-MS. Gas chromatographic conditions (6890N, Shanghai Wansai Technology Co., Shanghai, China): hydrogen flame ionization detector (FID), DB-FastFAME column; inlet temperature 250 °C, split ratio 20:1; detector temperature 260 °C; column initial temperature 80 °C, held for 0.5 min; ramped up to 165 °C with 40 °C/min programs, held for 1 min; ramped up to 230 °C with 4 °C/min programs. In addition the column was maintained at this temperature for 4 min.

### 2.10. Sodium Dodecyl Sulfate Polyacrylamide Gel Electrophoresis (SDS-PAGE)

Egg yolk samples after ozone treatment (0 min, 10 min, 20 min, 30 min, and 40 min) were measured using an electrophoresis instrument (DYY-12, Beijing Liuyi Instrument Factory, Beijing, China). The egg yolk solution was prepared into a sample solution with a protein concentration of 2 mg/mL, 80 μL of the solution was taken into a 200 μL small centrifuge tube, 20 μL of protein loading buffer was added, mixed, and heated in a boiling water bath for 3–5 min, cooled to room temperature and set aside. The concentration of isolated gel was 12%, the concentration of concentrated gel was 5%, and the electrode buffer system was a buffer solution of 0.05 mol/L Tris-HCl, 0.1% SDS, and 0.384 mol/L glycines. Samples were loaded with 15 μL and gel electrophoresis was performed under constant flow conditions at 80 V in the concentrated gel and increased to 120 V after entering the separation gel. Gel elution was performed in a flat dish, fixation solution was fixed for 30 min, the staining solution was stained for 30 min, and then decolorization was performed. Information was acquired using a gel imaging system (GelDoc XR+, Bio-Rad Corporation, Hercules, CA, USA).

### 2.11. Scanning Electron Microscope (SEM)

After the treatments in Section 2.3, approximately 2 g of yolk samples were taken and fixed with 2.5% glutaraldehyde for 8 h, then eluted with ethanol gradient solutions (60%, 70%, 80%, 90%, and 100%) and finally lyophilized. Each dried sample was sprayed with gold. Microstructures were observed and recorded using a scanning electron microscope (JSM-6390L V, NTC Corporation, Tokyo, Japan) at an accelerating voltage of 10 kV in low vacuum mode.

### 2.12. Data Analysis

All experiments were performed in 3 parallel groups, and the results were expressed as the mean of standard deviation (SD). An analysis of variance (ANOVA) was performed on the data using IBM SPSS Statistics 19.0 software (IBM Corporation, New York, NY, USA), and the means were compared by Tukey’s honest significant difference (HSD) test using a 5% level of significance. The quantitative extraction of peak areas of volatiles was uniformly deconvoluted by the automated mass spectrometry deconvolution and identification system (National Institute of Standards and Technology, Gaithersburg, MD, USA) to eliminate matrix effects of target volatiles and to extract pure chromatographic and mass spectrometric peaks. Cluster analysis was completed through the LianChuan BioCloud platform (https://www.omicstudio.cn, accessed on 10 September 2022).

## 3. Results and Discussion

### 3.1. Volatile Substance Analysis

As shown in Figure S1, the main peak shapes and peak emergence times of the GC-MS spectra of egg yolk remained the same under different ozone treatment times. Table S1 showed that a total of 18 volatile components were identified in the egg yolk from five groups with different ozone treatment times. Quantities of 18, 17, 16, 14, and 14 volatiles were identified from egg yolks treated with ozone for 0 min, 10 min, 20 min, 30 min, and 40 min, respectively. The identified 18 volatiles were n-hexane, hexadecane, 2-nonen-1-ol, cedrene, pentanal, hexanal, heptanal, octanal, 2,5-dihydroxybenzaldehyde, nonanal, decanal, undecanal, 6-methyl-5-hepten-2-one, geranyl acetone, 3-methyl-1-butanol, nonen-1-ol, 2-cyanoacetamide, and dibutyl amine. Nonanal and octanal accounted for 20.48% and 27.35% of the yolk volatiles and were the main contributing components to the yolk’s flavor [17]. The identified egg yolk volatiles were mainly classified into alkanes, terpenes, aldehydes, alcohols, ketones, and amines, among which aldehydes and alcohols accounted for a higher proportion [18]. In addition, it could be seen from Table S1 that the types of yolk volatiles decreased significantly (*p* < 0.05) with the increase in ozone treatment time, and the types of yolk volatiles decreased by 5.6%, 11.1%, 22.3%, and 22.3% after 10 min, 20 min, 30 min and, 40 min of ozone treatment, respectively.

Gouda et al. [18] explored the effect of bioactive terpenes on egg yolk volatiles, and a total of 111 different volatile compounds were identified by using GC-MS. The addition of bioactive terpenes significantly (*p* < 0.05) reduced the content of some ketones, amines, and nitro and organic acid compounds and thus improved the flavor of egg yolks [18]. It is noteworthy that the contents of hexane, hexadecane, heptanal, and dibutyl amine decreased significantly (*p* < 0.05) with the increase in ozone treatment time. No alkane volatiles were detected in the egg yolk after 40 min and 30 min of ozone treatment, while the content of aldehydes in the egg yolk increased significantly (*p* < 0.05). Egg yolks produced the highest content and the most diverse range of volatiles in aldehydes. Aldehydes had a low threshold and made an important contribution to flavor [19]. The highest content of nonanal had a strong oily and sweet orange odor. And its ethanolic solution had a vanillin odor, which is used as a food additive, and is also used in the preparation of rose, orange blossom, fragrant violet, and scented flavors [20]. The odor of nonanal played a dominant role in the egg yolk’s volatiles.

**Figure 1:**
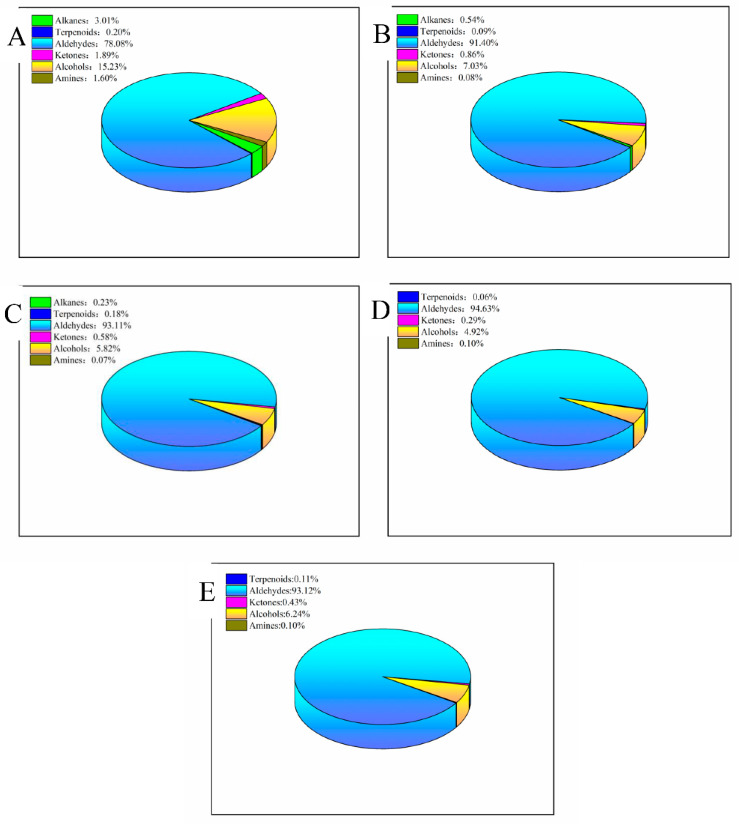
Distribution of volatile species and relative content of egg yolk under different ozone treatment times. (**A**) 0 min; (**B**) 10 min; (**C**) 20 min; (**D**) 30 min; (**E**) 40 min.

Figure 1 showed that the percentages of aldehydes in egg yolk treated with ozone for 0 min, 10 min, 20 min, 30 min, and 40 min were 78.08%, 91.40%, 93.11%, 94.63%, and 93.12%, respectively. It could be assumed that ozone oxidation promoted the degradation of fatty acids in egg yolks, thus producing more aldehyde volatiles. The aldehyde volatiles had a certain fruit flavor, so with the increase in aldehydes substantially improved the flavor of egg yolks [21]. In addition, it was found that the content of dibutyl amine, which had a strong ammonia taste and irritation, decreased in the yolk with the increase in ozone treatment time. It could be concluded that ozone treatment might be beneficial to improving the effects of undesirable flavors in the yolk.

### 3.2. Comparative Analysis of Egg Yolk Volatiles

**Figure 2:**
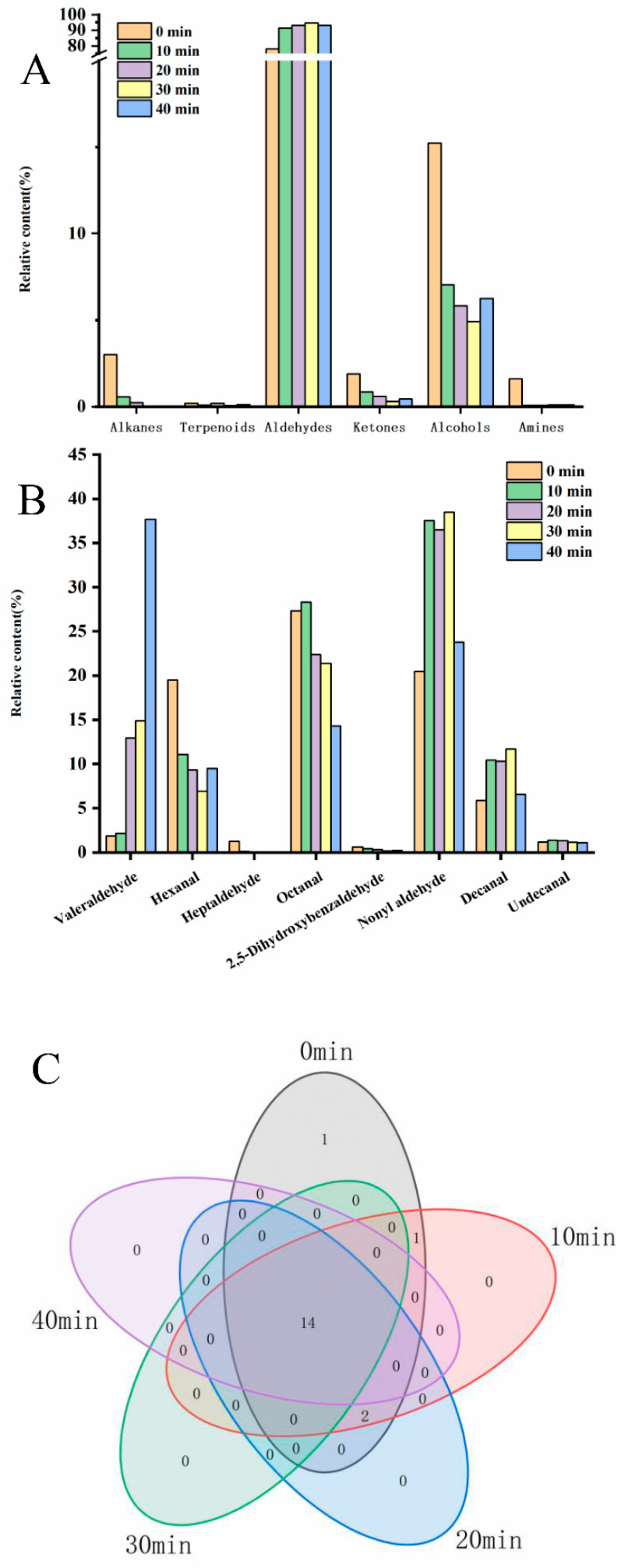
Composition of egg yolk volatile fraction species under different ozone treatment times. (**A**) Total volatiles; (**B**) aldehyde volatiles; (**C**) Wayne diagram.

In Figure 2A, it could be seen that the volatile substances in egg yolk were mainly alkanes, terpenes, aldehydes, ketones, alcohols, and amines. The content of alkane compounds decreased with increasing ozone treatment time, and no volatile alkane compounds were detected after 30 min and 40 min of ozone treatment time. The content of terpenoids in the egg yolk remained low and did not change crucially with the increase in treatment time. In addition, aldehydes and alcohols could be found as the main contributing components of egg yolk volatiles [22]. Among them, the content of aldehydes produced a certain degree of increase with the prolongation of ozone treatment time, while the alcohol compounds showed a decreasing trend. The content of ketones and amines in the egg yolk also had a certain degree of reduction with increasing treatment time. Ozone acted as an oxidizing agent to induce oxidative degradation of some compounds in the egg yolk that were susceptible to oxidation [23]. The decrease in alkanes, alcohols, ketones, and amines indirectly reflects the ozone attack on these compounds under the oxidation of ozone, which in turn triggers their conversion to other oxidation products [24].

Since aldehyde volatiles were the main egg yolk volatiles, the mechanism of ozone action on egg yolk volatile substances was investigated by analyzing the classification and relative content of aldehydes. Figure 2B shows the classification and relative contents of egg yolk aldehydes volatiles under different ozone treatment times. Valeraldehyde, hexanal, heptanal, octanal, 2,5-dihydroxybenzaldehyde, decanal, undecanal and nonanal were the main components of egg yolk aldehyde volatiles, among which nonanal and octanal had the highest contents. In addition, it was found that the content of hexanal and octanal in the egg yolk showed a decreasing trend with the increase in ozone treatment time. However, a significant increase in the relative content of glutaraldehyde occurred with increasing treatment time. Aldehyde volatiles were also transformed after ozone treatment, and it is assumed that aldehydes with longer carbon chains in the yolk were attacked by oxidation, resulting in shorter carbon chains [25]. As shown in Figure 2C, a Venn diagram was used for the classification of egg yolk volatiles at different ozone treatment times, and 14 volatiles were identified collectively from the five treatment groups. One volatile substance was identified with the control group by ozone treatment for 10 min, and one volatile substance was identified with the control group by ozone treatment for 20 min. Overall, ozone treatment reduced unfavorable volatiles, such as amines, which tended to have a negative impact on the flavor of egg yolks [26]. In addition, ozone did not destroy components with a relatively large contribution of volatiles and also increased the relative content of aldehyde volatiles. Low molecular weight aldehydes had an unpleasant odor, but as the molecular weight of the aldehyde increased, fruit flavors began to emerge [27]. The content of aldehydes with long carbon chains in the egg yolk increased substantially after ozone treatment (10 min, 20 min, and 30 min), thus contributing to the improvement of the egg yolk’s flavor. Egg yolks contain the highest relative proportion and variety of aldehyde volatiles [26]. Therefore, aldehyde volatiles make an important contribution to the flavor of egg yolks. The main reason for the increase in aldehyde volatiles could be the oxidation of fatty acids caused by ozone treatment.

### 3.3. Principal Component Analysis of Egg Yolk Volatiles

**Figure 3:**
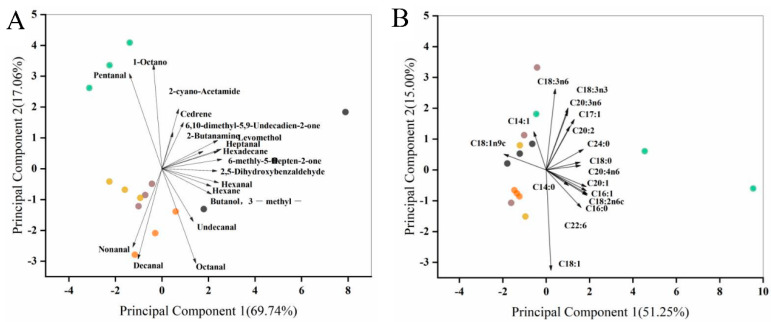
Principal component analysis and loadings of volatile compounds and fatty acids of egg yolk under different ozone treatment times. (**A**) The volatiles; (**B**) fatty acids. Black, green, yellow, purple, and orange spheres represent three parallel groups of samples treated with ozone for 0 min, 10 min, 20 min, 30 min, and 40 min.

Principal component analysis is one of the most widely used multivariate mathematical and statistical methods [28]. Figure 3A shows the principal component analysis (PCA) of egg yolk volatiles at different ozone treatment times, and the differences between the groups were assessed based on the contribution of the PC factors. The first and second principal components accounted for 69.74% and 17.06% of the total variance, respectively. Table S2 shows the characteristic values and contribution of the 18 volatile substances. Based on the principle that the eigenvalues were greater than 1, a total of four principal components were analyzed, with contributions of 69.74%, 17.06%, 13.06%, and 0.14%, respectively. The cumulative contribution of the first two principal components reached 86.80%, and they represented the volatile substances of egg yolk samples well.

As can be seen from Table S3, PC1 had high loading coefficients in the variables pentanal, cedrene, nonanal, and dibutyl amine, indicating that these variables had a high correlation with PC1. PC2 had high loading coefficients in the variables undecanal, glutaraldehyde, 2-cyano-acetamide, and cedrene, indicating that these variables were highly correlated with PC2. Table S2 shows that 86.07% of the total variance was contributed by PC1 and PC2. The largest contribution to PC1 was made by glutaraldehyde and cedrene, and the largest contribution to PC2 was made by undecylenic aldehyde. Therefore, valeraldehyde and undecylenic aldehyde were regarded as volatile substances that had a major impact on the flavor and texture of the egg yolks. Figure 3A shows the principal component analysis and loadings of egg yolk volatiles at different ozone treatment times. It could be found that the control and 10 min ozone-treated samples were located in quadrant IV and quadrant II, respectively, while the volatiles of 20 min, 30 min, and 40 min ozone-treated egg yolk samples were located in quadrant III. In short, the nonanal, decanal, and octanal were the main egg yolk volatiles after ozone treatment (10 min, 20 min, and 30 min).

### 3.4. Cluster Analysis of Egg Yolk Volatiles

**Figure 4:**
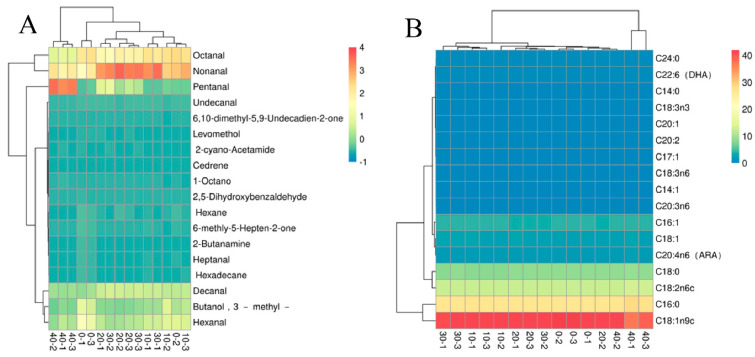
Heat map of volatiles and fatty acids of egg yolk at different ozone treatment times. (**A**) The volatiles; (**B**) fatty acids.

As a method of visualizing and analyzing the distribution of experimental data, heat maps can used for quality control of experimental data and visualization of discrepant data, as well as for clustering data and samples to observe differences in samples [29]. A total of 18 volatile flavor substances were identified from the egg yolk liquor of the five ozone-treated groups by SPME-GC-MS analysis combined with compound database comparison. These included two alkanes, two terpenoids, eight aldehydes, two ketones, two alcohols, and two amines. Immediately after, it was the thermograms of egg yolk volatiles for different ozone treatment times was drawn through an online website, as shown in Figure 4A. The size of the difference between the mean values of each component in the graph is indicated by different shades of color, the darker the color is, the more content it has, and vice versa. The darkest color was the unique component among different species [30]. The results of the thermogram also indicated that glutaraldehyde, hexanal, octanal, nonanal, decanal, and 3-methyl-1-butanol were the main volatiles in the egg yolk. The 20 min and 30 min ozone oxidation treatment groups showed higher levels of nonanal than the control group. In summary, moderate ozone treatment (20 min and 30 min) of egg yolk liquid yielded increased aldehyde volatiles, which in turn might contribute to a better flavor of the yolk.

### 3.5. Fatty Acid Analysis

Table S4 showed the fatty acid composition and relative contents of egg yolk under different ozone treatment times, and 17 fatty acids were identified. Among them, oleic acid and palmitic acid were the major fatty acids in the egg yolk, accounting for 41.47% and 26.89% of the total fatty acids in the untreated egg yolk, respectively [31]. Myristic acid, myristic brain acid, *cis*-10-heptadecenoic acid, γ-linolenic acid, α-linolenic acid, *cis*-11-eicosatrienoic acid, *cis*-8,11,14-eicosatrienoic acid, carnosic acid and docosahexaenoic acid were the relatively small amounts of fatty acids in egg yolk, accounting for 0.34%, 0.09%, 0.08%, 0.11%, 0.27%, 0.14%, 0.14%, 0.63%, and 0.56% of the total fatty acids in the egg yolk, respectively. No significant changes were found in the types of yolk fatty acids after ozone treatment, but the relative contents of the components differed significantly (*p* < 0.05). Notably, *cis*-11,14-eicosadienoic acid was not detected in untreated egg yolks but was detected in all ozone-treated egg yolks. In addition, there was a significant difference in the relative content of palmitic acids in the treated group compared to the control group. This might be due to the oxidation of ozone that disrupted the long-chain fatty acids in the yolk and thus part of the carbon chain breaks, causing an increase in the content of these fatty acids [32]. Oleic acid, the major fatty acid in egg yolk, underwent a significant decrease after 40 min of ozone treatment. 

The relative content of linoleic acid in egg yolk increased significantly (*p* < 0.05) with the increase in ozone treatment time. The first unsaturated double bond of oleic acid was located on the penultimate ninth carbon atom at the end of the carbon chain (methyl ester end), which was an ω-9 type polyunsaturated fatty acid. The first unsaturated double bond of linoleic acid was located at the penultimate sixth carbon atom at the end of the carbon chain (methyl ester end), which was an ω-6-type polyunsaturated fatty acid. In addition, linoleic acid was more easily absorbed by the body than oleic acid [33].

### 3.6. Principal Components and Clustering Analysis of Egg Yolk Fatty Acids

The principal component analysis of egg yolk fatty acids at different ozone treatment times, and the differences between the groups were assessed based on the contribution of the PC factors (Figure 3B). Among them, the first and second principal components accounted for 51.25% and 15.00% of the total variance, respectively. Table S5 showed the eigenvalues and contribution rates of the 17 detected fatty acids. Based on the principle of eigenvalues greater than 1, a total of four principal components were analyzed, with contribution rates of 51.25%, 15.00%, 11.57%, and 7.55%, respectively [34]. The cumulative contribution of the first two principal components reached only 66.25%, while the cumulative contribution of the first four principal components exceeded 85%. 

PC1 had high loading coefficients in the variables *cis*-11,14-eicosadienoic acid and arachidonic acid, suggesting that these variables had a high correlation with PC1 (Table S6). PC2 had high loading coefficients in the variables isoleic acid, linoleic acid, and *cis*-8,11,14-eicosatrienoic acid, indicating a high correlation between these variables and PC2. As shown in Table S5, 66.25% of the total variance was contributed by PC1 and PC2, with the largest contribution to PC1 being *cis*-11,14-eicosadienoic acid and the largest contribution to PC2 being isoleic acid. Figure 3B showed the principal component analysis and loadings of fatty acids of egg yolks at different ozone treatment times. It could be found that the control and 10 min ozone-treated samples were located in the fourth and second quadrants, respectively, while the fatty acids of egg yolk samples treated with ozone for 20 min, 30 min, and 40 min were located in the second and third quadrants. After ozone treatment, the yolk’s fatty acids that played a major role in the principal component analysis were oleic acid, isoleic acid, and myristic acid. The thermograms of fatty acids in egg yolk at different ozone treatment times are shown in Figure 4B, and it can be seen that oleic, palmitic, linoleic, and stearic acids were the dominant fatty acids in the egg yolk. The relative contents of isoleic acid and arachidonic acid were closer.

### 3.7. SDS-PAGE Analysis

**Figure 5:**
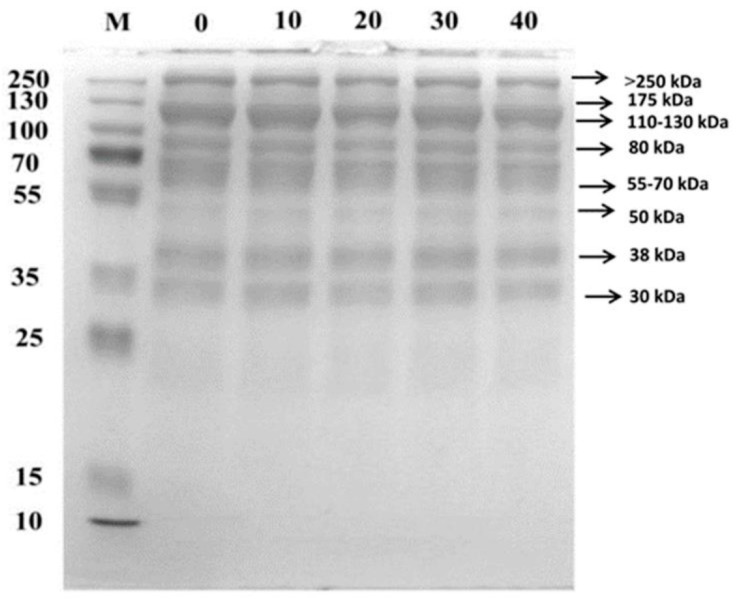
Electropherograms of SDS-PAGE of egg yolk under different ozone treatment times.

The electrophoresis of egg yolk at different ozone treatment times was shown in Figure 5. Egg yolk proteins were mainly divided into high-density lipoprotein (HDL), low-density lipoprotein (LDL), immunoglobulin yolk (IgY), and phosvitin [35]. LDL of egg yolk was mainly divided into 5 de-cofactorized proteins and 6 apolipoproteins. The egg yolk HDL was a dimeric protein, consisting of two globulins and five apolipoproteins. A lighter protein band located around 175 kDa could be observed in Figure 5, which might be one of the apolipoproteins of LDL. A darker colored protein band was present between 110–130 kDa, which was presumed to be mainly the two subunits of HDL and β-HDL. A weaker protein band was present near 80 kDa, which corresponded to the apolipoprotein with the highest molecular weight in HDL. Multiple bands were present between 55–70 kDa for egg yolk proteins, which were probably mainly several de-cofactored proteins of LDL. The protein bands located around 50 kDa and 30 kDa corresponded to the two smaller molecular weight HDL apolipoproteins, respectively. The results of SDS-PAGE of egg yolk showed no significant difference among the groups. It was presumed that aggregation of egg yolk proteins probably occurred under excessive oxidation, but this aggregation was weak and not observed in the SDS-PAGE results, which was probably because the treatment volume and ozone flow rate limited the extent of oxidation. In addition, a darker protein band was observed in the region larger than 250 kDa in Figure 5, which presumably was a larger protein molecule in the yolk. In addition, it could be assumed that the oxidation-induced aggregation behavior did not cause a significant change in the intensity of the molecular band at a larger yolk protein molecular weight.

### 3.8. SEM Analysis

**Figure 6:**
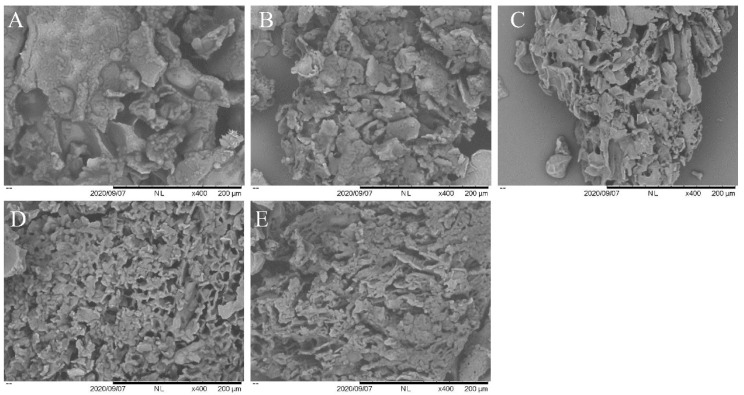
The effect of different ozone treatment time on the microstructure of chicken egg yolk powder. (**A**) 0 min; (**B**) 10 min; (**C**) 20 min; (**D**) 30 min; (**E**) 40 min.

Figure 6 showed the electron micrographs of the egg yolk samples treated with ozone for 0 min, 10 min, 20 min, 30 min, and 40 min in that order. As seen from the SEM images, the control group of lyophilized egg yolk powder had larger masses and a single spatial structure. However, with the extension of ozone treatment time, the structure of the lyophilized powders in the 10 min and 20 min treatment groups gradually changed to disordered folding and the spatial network structure increased substantially. The freeze-dried egg yolk powder treated with ozone for 30 min had more pores and the structure became denser with a more uniform pore size distribution. The yolk powder structure was more disordered in the 40 min treatment group compared to the 30 min treatment group. Therefore, it could be speculated that ozone treatment improved the flexibility of egg yolk proteins and affected the conformation and interaction of proteins, which in turn drove the egg yolk powder structure towards a denser network structure. However, as the intensity of ozone treatment was further increased, some proteins of the yolk triggered aggregation behavior under the effect of excessive oxidation, resulting in aggregates that affected the structure of the yolk powder. The loose and porous network structure of egg yolk powder was thought to be closely linked to better functional properties, such as solubility [36]. In summary, moderate ozonation treatment (20 min and 30 min) obviously improved the structure of egg yolk powder and has the potential to improve the properties of egg yolk powder.

## 4. Conclusions

In summary, this study investigated the effect of ozone treatment on egg yolk volatiles and fatty acids. It was found that moderate ozone treatment (20 min and 30 min) might help improve the flavor of egg yolk. Aldehydes were significantly increased in the ozone-treated group, and dibutyl amine was not detected after ozonation, indicating that ozone played a positive role in the improvement of flavor substances in egg yolk. There was no significant difference in fatty acid species between the yolks of the ozone-treated and control groups, but their relative contents differed. It is worth noting that the double bonds of monounsaturated fatty acids in egg yolks were broken after ozone treatment, and then saturated fatty acids were produced. The SEM results showed that the network structure of the yolk was regular and dense in the 30 min treatment group. The results of SDS-PAGE showed no significant differences among the groups of yolk proteins. 

Egg yolk was widely used in baked goods, full-value nutritional formulas and confectionery due to its excellent emulsification and gelation properties and the raw materials for further processed egg products are mainly egg liquor or egg powder. However, homogenization, filtration, and sterilization in egg yolk processing could cause a certain degree of loss to its functional properties and nutritional value, so it is important to use modification techniques (ozone-mediated oxidation) to improve functional properties and flavor and acts as sterilization of egg yolk liquor in the processing of egg yolk products. These findings expanded upon and provided a theoretical basis for the application of ozone modification technology in the egg yolk processing industry.

## Supplementary Materials

The following supporting information can be downloaded at: https://www.mdpi.com/article/10.3390/molecules28010124/s1, Figure S1: Total ion flow of egg yolk volatiles under different ozone treatment times. A, 0 min; B, 10 min; C, 20 min; D, 30 min; E, 40 min; Table S1: Fraction and relative content of volatile substances in egg yolk under different ozone treatment times; Table S2: Eigenvalues and contribution rates of principal components; Table S3: Principal component loading matrix; Table S4: Fatty acid composition and relative content of egg yolk under different ozone treatment times; Table S5: Eigenvalues and contribution rates of principal components; Table S6: Principal component loading matrix.
