# Supplementary material for: An Efficient Processing Strategy to Improve the Flavor Profile of Egg Yolk: Ozone-Mediated Oxidation
Source: Molecules. 2022 Dec 23;28(1):124. doi: 10.3390/molecules28010124 (PMC9822375; doi:10.3390/molecules28010124)
Supplement: Supplementary file 1 [file molecules-28-00124-s001.zip › molecules-1990058-supplementary.pdf]

Supplementary Data

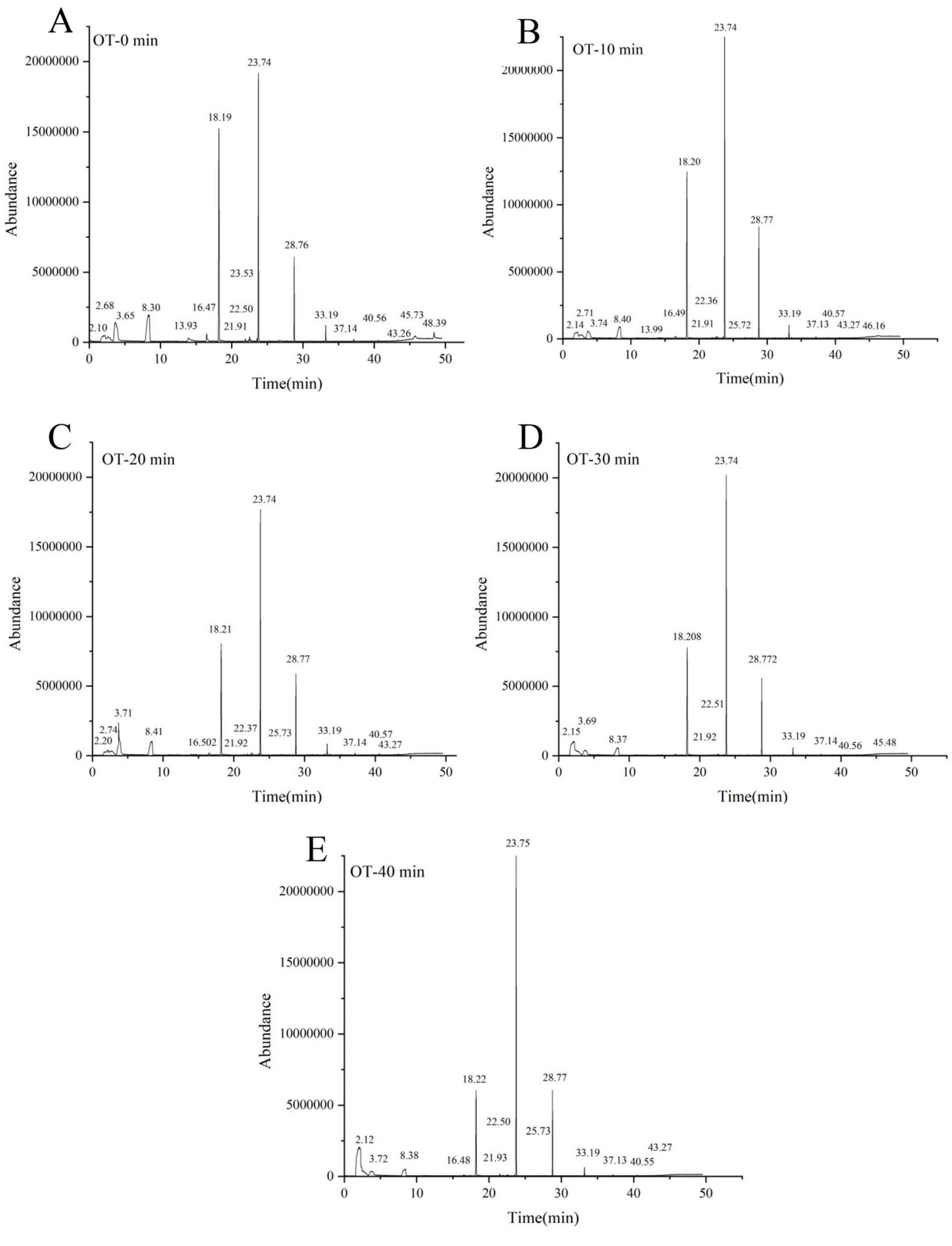

Figure S1. Total ion flow of egg yolk volatiles under different ozone treatment times.

A, 0 min; B, 10 min; C, 20 min; D, 30 min; E, 40 min.

Table S1. Fraction and relative content of volatile substances in egg yolk under different ozone treatment times

Peak number	Volatile compounds	RT*	Relative content of volatiles (%)				
			0 min	10 min	20 min	30 min	40 min
	Alkanes						
1	Hexane	2.68	1.73±0.24[a]	0.22±0.14[b]	0.21±0.12[b]	nd	nd
2	Hexadecane	45.73	1.28±0.29[a]	0.32±0.13[b]	0.02±0.02[c]	nd	nd
	Total content		3.01	0.54	0.23	–	–
	Terpenoids						
3	Levomethol	26.67	0.15±0.06[a]	0.04±0.00[c]	0.13±0.07[a]	0.02±0.01[c]	0.07±0.04[b]
4	Cedrene	43.26	0.05±0.00[a]	0.05±0.03[a]	0.05±0.02[a]	0.04±0.03[a]	0.04±0.00[a]
	Total content		0.20	0.09	0.18	0.06	0.11
	Aldehydes						
5	Pentanal	2.10	1.90±1.15[e]	2.18±1.36[d]	12.95±4.15[c]	14.90±8.93[b]	37.64±2.40[a]
6	Hexanal	8.30	19.47±4.73[a]	11.11±1.26[b]	9.35±1.35[c]	6.90±2.94[d]	9.49±2.43[c]
7	Heptanal	13.93	1.24±0.65	0.14±0.10	nd	nd	nd
8	Octanal	18.19	27.35±1.57 [b]	28.28±0.38[a]	22.38±1.75[c]	21.35±3.61[d]	14.30±1.00[e]
9	2,5-Dihydroxybenzaldehyde	22.36	0.61±0.13[a]	0.44±0.23[b]	0.34±0.01[c]	0.19±0.08[e]	0.24±0.04[d]
10	Nonanal	23.73	20.48±7.78[e]	37.49±4.69[b]	36.50±3.79[c]	38.49±1.62[a]	23.80±2.97[d]
11	Decanal	28.76	5.88±2.5[e]	10.42±0.8[b]	10.29±0.57[c]	11.68±1.02[a]	6.56±0.32[d]
12	Undecanal	33.19	1.15±0.24[c]	1.35±0.24[a]	1.31±0.19[b]	1.12±0.01[d]	1.08±0.23[e]
	Total content		78.08	91.40	93.11	94.63	93.12
	Ketones						
13	6-methly-5-Hepten-2-one	16.47	1.72±0.54[a]	0.71±0.46[b]	0.44±0.16[c]	0.16±0.02[e]	0.26±0.09[d]
14	6,10-dimethyl-5,9-Undecadien-2-one	37.14	0.17±0.01[a]	0.15±0.07[b]	0.14±0.06[b]	0.13±0.05[c]	0.17±0.04[a]
	Total content		1.89	0.86	0.58	0.29	0.43

(Continued Table 1)

Peak number	Volatile compounds	RT*	Relative content of volatiles (%)				
			0 min	10 min	20 min	30 min	40 min
	Alcohols						
15	Butanol,3-methyl-	3.65	15.00±4.84[a]	6.88±2.44[b]	5.62±1.00[c]	4.75±1.46[d]	5.62±1.53[c]
16	1-Octano2-	21.91	0.23±0.05[b]	0.14±0.09[d]	0.21±0.06[b]	0.17±0.05[c]	0.63±0.25[a]
	Total content		15.23	7.03	5.82	4.92	6.24
	Amines						
17	2-cyano-Acetamide	40.56	0.10±0.02[a]	0.08±0.02[a]	0.07±0.00[b]	0.10±0.01[a]	0.10±0.02[a]
18	dibutyl amine	48.39	1.50±0.74[a]	nd	nd	nd	nd
	Total content		1.60	0.08	0.07	0.10	0.10
	Number of compounds		18	17	16	14	14

nd indicates not detected; RT * is retention time; different letter superscript indicates significant difference between groups (P < 0.05)

Table S2. Eigenvalues and contribution rates of principal components

Main Factors	Eigenvalue	Variance contribution rate (%)	Cumulative contribution rate (%)
1	8.40601	69.74 %	69.74%
2	4.14660	17.06%	86.80%
3	2.35016	13.06%	99.86%
4	1.48831	0.14%	100.00%
5	0	0.00%	100.00%
6	0	0.00%	100.00%
7	0	0.00%	100.00%
8	0	0.00%	100.00%
9	0	0.00%	100.00%
10	0	0.00%	100.00%
11	0	0.00%	100.00%
12	0	0.00%	100.00%
13	0	0.00%	100.00%
14	0	0.00%	100.00%
15	0	0.00%	100.00%
16	0	0.00%	100.00%
17	0	0.00%	100.00%
18	0	0.00%	100.00%

Table S3. Principal component loading matrix

Volatile substances	Principal Components	
	1	2
Pentanal	7.89131	1.83752
Hexane	1.80016	-1.30811
Butanol,3-methyl-	4.84574	0.26478
Hexanal	0.59059	-1.38859
Heptanal	-1.16173	-2.78698
6-methly-5-Hepten-2-one	-0.28564	-2.08791
Octanal	-0.42900	-0.48850
1-Octano	-1.00453	-1.21469
2,5-Dihydroxybenzaldehyde	-0.71676	-0.85156
Nonanal	4.92833	-0.94592
Levomethol	-2.25604	-0.41655
Decanal	-1.59219	-0.68122
Undecanal	-1.38154	4.09136
6,10-dimethyl-5,9-Undecadien-2-one	-3.12083	2.62050
2-cyano-Acetamide	-2.25122	3.35588
Cedrene	7.89131	1.83752
Hexadecane	1.80016	-1.30811
2-Butanamine	4.84574	0.26478

Table S4. Fatty acid composition and relative content of egg yolk under different ozone treatment times

Fatty acid (%)	0 min	10 min	20 min	30 min	40 min
C14:0	0.34±0.01^{a}	0.31±0.02^{a}	0.33±0.00^{a}	0.33±0.01^{a}	0.35±0.02^{a}
C14:1	0.09±0.01^{b}	0.08±0.01^{a}	0.08±0.01^{a}	0.09±0.00^{a}	0.08±0.01^{a}
C16:0	26.89±0.04^{b}	27.06±0.09^{ab}	26.93±0.10^{b}	27.11±0.21^{ab}	28.76±1.37^{a}
C16:1	3.84±0.01^{b}	3.86±0.01^{ab}	3.80±0.01^{b}	3.84±0.01^{b}	4.08±0.16^{a}
C17:1	0.08±0.05^{a}	0.00±0.00^{b}	0.00±0.00^{b}	0.00±0.00^{b}	0.11±0.01^{a}
C18:0	8.52±0.03^{b}	8.26±0.04^{b}	8.60±0.05^{b}	8.40±0.14^{b}	9.14±0.42^{a}
C18:1n9c	41.47±0.15^{a}	41.53±0.22^{a}	41.42±0.09^{a}	41.07±0.26^{b}	37.01±3.18^{c}
C18:1	2.36±0.04^{b}	2.40±0.19^{ab}	2.56±0.22^{ab}	2.86±0.27^{a}	2.58±0.19^{ab}
C18:2n6c	12.53±0.02^{b}	12.58±0.01^{ab}	12.50±0.02^{b}	12.57±0.09^{ab}	13.36±0.60^{a}
C18:3n6	0.11±0.07^{a}	0.12±0.08^{a}	0.00±0.07^{b}	0.00±0.00^{b}	0.12±0.01^{a}
C18:3n3	0.27±0.01^{a}	0.27±0.02^{a}	0.28±0.01^{a}	0.25±0.02^{a}	0.29±0.00^{a}
C20:1	0.14±0.01^{b}	0.15±0.10^{b}	0.14±0.01^{b}	0.15±0.00^{b}	0.30±0.11^{a}
C20:2	0.00±0.00^{c}	0.25±0.10^{a}	0.26±0.01^{a}	0.17±0.09^{b}	0.24±0.04^{a}
C20:3n6	0.14±0.09^{a}	0.00±0.00^{b}	0.00±0.00^{b}	0.00±0.00^{b}	0.17±0.02^{a}
C20:4n6	2.03±0.00^{b}	2.04±0.00^{b}	2.03±0.01^{b}	2.05±0.03^{b}	2.15±0.01^{a}
C24:0	0.63±0.00^{a}	0.51±0.01^{b}	0.52±0.01^{b}	0.54±0.01^{b}	0.65±0.01^{a}
C22:6	0.56±0.01^{b}	0.58±0.01^{ab}	0.56±0.00^{b}	0.57±0.01^{ab}	0.60±0.02^{a}

Different letter superscripts indicate significant differences between groups ($P < 0.05$).

Table S5. Eigenvalues and contribution rates of principal components

Main Factors	Eigenvalue	Variance contribution rate (%)	Cumulative contribution rate (%)
1	9.22447	51.25%	51.25%
2	2.69970	15.00%	66.25%
3	2.08240	11.57%	77.81%
4	1.35853	7.55%	85.36%
5	0.93095	5.17%	90.53%
6	0.79928	4.44%	94.97%
7	0.59058	3.28%	98.26%
8	0.17929	1.00%	99.25%
9	0.13480	0.75%	100.00%
10	0	0.00%	100.00%
11	0	0.00%	100.00%
12	0	0.00%	100.00%
13	0	0.00%	100.00%
14	0	-0.00%	100.00%
15	0	-0.00%	100.00%
16	0	-0.00%	100.00%
17	0	-0.00%	100.00%

Table S6. Principal component loading matrix

Fatty acid	Principal Components	
	1	2
C14:0	-0.64321	0.84859
C14:1	-1.79145	0.20950
C16:0	-1.21733	0.52905
C16:1	-1.23354	-0.85722
C17:1	-1.46682	-0.65688
C18:0	-1.35018	-0.75705
C18:1n9c	-1.61156	-1.06749
C18:1	-0.42122	3.32565
C18:2n6c	-1.01639	1.12908
C18:3n6	-0.70039	-3.81897
C18:3n3	-1.21460	0.79976
C20:1	-0.95749	-1.50960
C20:2	9.54032	-0.60066
C20:3n6	-0.45754	1.81770
C20:4n6	4.54139	0.60852
C24:0	-0.64321	0.84859
C22:6	-1.79145	0.20950
